# Primary health care strengthening through the lens of healthcare system thinking

**DOI:** 10.4102/safp.v67i1.6039

**Published:** 2025-01-23

**Authors:** Ramprakash Kaswa, Klaus von Pressentin

**Affiliations:** 1 Department of Family Medicine and Rural Health, Faculty of Health Sciences, Walter Sisulu University, Mthatha, South Africa; 2 Department of Family, Community and Emergency Care, Faculty of Health Sciences, University of Cape Town, Cape Town, South Africa

**Keywords:** healthcare system, primary care, family physician, clinical governance, leadership

## Abstract

Despite the strides made in healthcare, many countries still struggle to meet citizen healthcare needs, leading to global and regional health inequalities. The complex interactions between healthcare systems and disciplines present challenges for primary care providers and family physicians. Primary care providers must be equipped with tools and resources to effectively fulfil their duties, such as clinical governance, leadership and capacity building. This article focusses on various thinking approaches that primary care providers can employ, namely systems thinking, complexity science thinking and learning health systems thinking. We appreciate that individual styles and preferences, organisational culture and systemic realities influence multiple modes of thinking and decision-making. A range of modes of thinking and mental models will assist with tackling challenges and opportunities in the primary healthcare system. We hope this brief overview encourages readers to experiment with different ways of thinking to help facilitate innovative solutions.

## Background

In a constantly changing world, health systems struggle to meet the dynamic healthcare needs of their citizens. Despite consistent progress in healthcare development, persistent inequity exists in people’s health status globally and within nations.^1^ South Africa is committed to achieving universal health coverage that aligns with global trends.^2,3^ According to Goal 3 of the sustainable development goals (SDGs), a high-quality primary health care system is essential for universal health coverage. The South African government recently promulgated the *National Health Insurance (NHI) Act* to give all citizens access to quality healthcare services regardless of socioeconomic status.^4^ In addition, it aims to give effect to the Constitution’s Section 27, which provides everyone the right to access quality health services.^5^ Thirty years after the country’s first democratic government was elected, South Africa is still working towards improving its primary health care system, facing numerous challenges.

**FIGURE 1:**
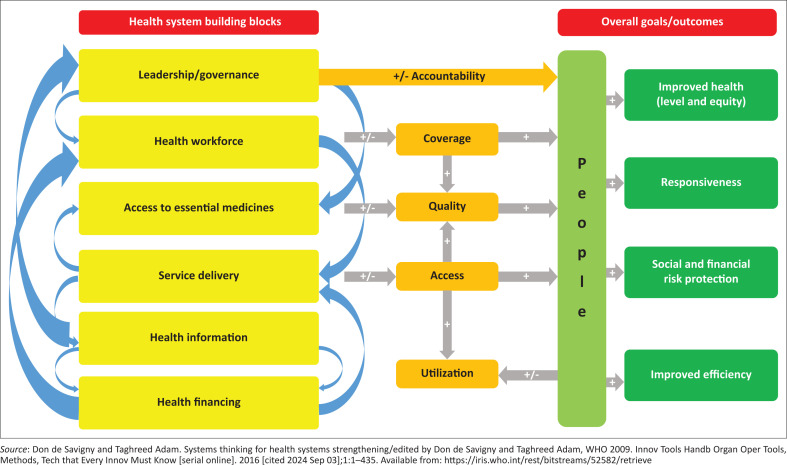
World Health Organization building block of healthcare system.

The structure of the healthcare system consists of various interlocking components designed to function dynamically and not in isolation. The World Health Organization (WHO) defined these components as the building blocks of the healthcare system. Figure 1 demonstrates the interconnectedness of healthcare system components. The health system can achieve its goals through the interactions and engagements among its components.^2,6,7^ Addressing the different forces within and across components or building blocks of the primary health care system is essential to achieving universal healthcare. A recent example of the dynamic forces influencing how the health system components interact is the coronavirus disease 2019 (COVID-19) pandemic, which demonstrated that a comprehensive approach to detecting and addressing emerging infectious conditions relies on responsive services with adequate coverage which can detect and contain infected individuals while strengthening the need for accountable leadership and financing models to govern vaccination rollout despite divergent socio-political forces affecting equitable access, as well differing sociocultural worldviews affecting vaccine utilisation. Highly effective country responses to the pandemic were in developing a healthcare systems resilience framework that includes preserving health systems functions and resources and reducing vulnerability.^8,9^

Family physicians and primary care providers are often challenged to deal with the interplay of these systemic forces as they manifest themselves in the primary care consultation and health service setting. These primary care clinicians should, therefore, be equipped to have a range of tools and ways of thinking to help them navigate their clinical and non-clinical roles, such as leadership and clinical governance, capacity building and advocating for equitable resource allocation in different platforms, such as the sub-district and district healthcare management form and when engaging with community stakeholders.^10,11,12^

## Introducing three thinking approaches

We will consider three approaches: systems thinking, complexity science thinking and learning health systems thinking. These approaches are not mutually exclusive or all-encompassing. Still, they will assist clinician-scholars, clinician-leaders and clinical governance champions with valuable tools to support their teams, patients and communities in finding a way forward. We will illustrate each thinking approach with examples linked to the primary care clinical setting to contextualise the learning experience.

## Systems thinking

The WHO views a healthcare system as consisting of ‘all organisations, people and actions whose primary intent is to promote, restore or maintain health’.^2^ At its heart, such a system should be geared towards ‘improving health and health equity in ways that are responsive, financially fair, and make the best, or most efficient, use of available resources’.^2^ Understanding this thinking approach will enable actors at various levels within the system, including healthcare workers, to leverage this healthcare system thinking approach to meet people’s healthcare needs.

The concept of systems thinking emphasises the importance of understanding the various components that make up the healthcare system. This allows them to identify the multiple factors affecting its efficiency and recognise the various component’s dependencies. This appreciation of the interconnectedness of the parts will allow an approach to organise an understanding of contributing forces and factors. Primary health care systems thinking aims to develop and implement inclusive healthcare systems.^6^ It involves conceptualising, planning and implementing a primary health care system within the larger framework of the healthcare service delivery platform. Designing, implementing and improving primary health care systems require an integrated and comprehensive approach.^13^ Systems thinking is helpful when dealing with issues related to clinical governance, such as performing root cause analysis during a morbidity and mortality meeting. It is also useful when visualising the macro-factors that may be influenced at a national or global level and may be considered in collective advocacy actions, such as access to essential medicines and motivating primary health care-orientated human resources for health policies. Instead of concentrating on individual aspects of the healthcare system, systems thinking aims to perceive it as a whole.^13^

## Complexity science thinking

This process can be very challenging because of the complexity of the healthcare system’s building block interdependency and nonlinear relationships. Furthermore, this complexity manifests differently when viewed from different system levels, as specific policy directions may make perfect sense from a national perspective. In contrast, the local realities at the clinical coalface draw these directions into question. New ways of thinking are needed to understand the characteristics of the healthcare system.^1^ These different thinking approaches must be a vital toolset for these dynamic complexities. Alternative modes of thinking may be relevant when considering contemporary challenges imposed by the contextual realities or the social determinants of health affecting the patient’s chances of achieving wellness in the consultation room.^14,15^ Here, at the micro-level of the health system, an appreciation of complexity science and complex adaptive systems theories may be needed, such as understanding the nonlinear interconnections between health and other sectors, such as food, trade and the environment. Appreciating complexity also helps with sense-making when appreciating the world and health services as a social system and how information interpretation and resultant behaviour may affect us individually and collectively.^16^

## Learning health systems thinking

Lastly, one should also embrace the concept of learning health systems or ecosystems, which encourages incorporating learning and lessons into growing an environment and fosters an appreciation of human factors that may result in adverse or desirable outcomes. This way of thinking facilitates leadership and systems development centred on building resilience and shaping organisational culture and design.^17^ Learning is fundamental to health systems strengthening and happens at different levels, individuals, teams, organisations and cross-organisations. This approach calls for an investment in developing a culture and capability of using locally generated knowledge to address local challenges. This approach to creating a learning health system will also facilitate an expansion from monitoring and evaluation (M&E), such as the approach used to monitor the South African district health system performance formally, to a more cohesive and integrated approach involving various stakeholders in the system in using data for decision-making at different levels and thereby ‘inculcating a learning approach’.^17,18^

Following this overview of thinking approaches, we will continue to explore health systems thinking as a way of appreciating the presiding ways of strengthening the systems in which we work and latching on to the terminology or ‘lingo’ frequently used by health service managers and policymakers. These thinking approaches encourage questioning or inquiry and foster problem-solving.

## How do these thinking approaches help develop a primary health care-orientated health system?

The WHO identifies quality care delivery as the first building block of systems thinking, emphasising the involvement of various stakeholders in designing and implementing health systems to align with the population’s priorities and needs.^6^ In 1997, a White Paper outlined the framework for establishing a unified primary health care system providing universal health coverage in South Africa.^1^ The first policy on quality in healthcare was published in 2001.^19^ It aims to unify healthcare quality goals, promote evidence-based decisions and ensure efficient healthcare system use. The *National Health Act of 2003* emphasises the government’s commitment to delivering quality healthcare consistently.^4^ However, the lack of a comprehensive regulatory framework remained a significant issue. In 2010, the National Department of Health (NDoH) reaffirmed its commitment to improving the healthcare system by implementing the 10-point plan to improve the sector. It also launched a service delivery agreement to enhance the quality of healthcare services.^5^ In 2012, the NDoH released a Quality Improvement Guide, which defines the various steps to deliver quality healthcare consistently.^17^ However, its implementation was hampered by the lack of proper planning and monitoring.^19^ In 2013, the NDoH launched the Ideal Clinic Realisation and Maintenance programme to improve primary health care and lay the foundation for implementing the NHI. The programme aimed to establish proper infrastructure, recruit staff and use information systems while developing the Integrated Clinical Services Management model to improve healthcare services quality.^20^

The health information and clinical governance subsystems are two crucial building blocks of a healthcare system. Missing information is considered the most common cause of system malfunctions, and inadequate clinical governance structures can contribute to poor performance.^21^ The 2017 Lancet National Commission noted that the lack of ethical leadership and governance in the healthcare system contributes to the poor quality of care in South Africa. The commission has identified four main recommendations that will help improve healthcare quality in South Africa. These include establishing effective leadership and governance, revitalising quality primary health care, investing in human resources and strengthening health information through effective monitoring and evaluation.^22^

## How can primary care practitioners, managers and teams leverage systems thinking to strengthen primary health care?

Primary health care is the foundation of a robust healthcare system. It refers to prevention, promotion and effective treatment of common medical conditions, rehabilitation and end-of-life assistance through palliative care.^13,23^ A high-quality primary health care system is vital to any community’s well-being. It ensures that all individuals have equal access to healthcare.

**FIGURE 2:**
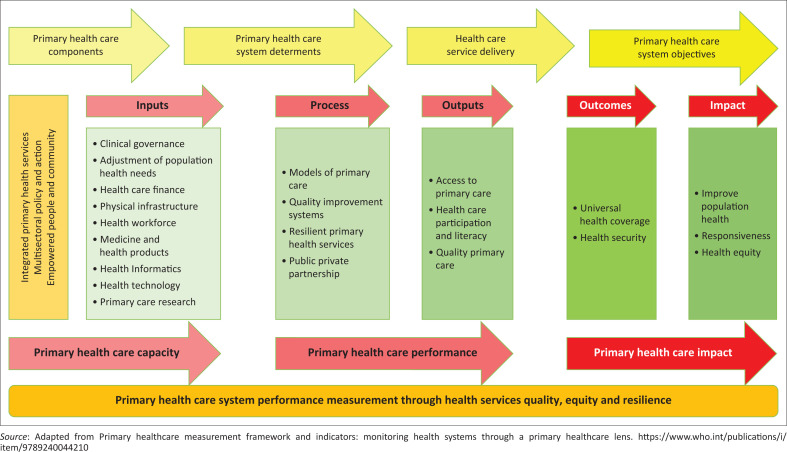
Conceptual framework primary health care theory of change.

As South Africa strives to improve the health of its citizens, it must be able to monitor and measure its progress. The WHO Primary Healthcare framework is based on the Theory of Change model, outlining how the primary care provider approach can achieve desired outcomes. It aims to connect primary health care components and desired outcomes, including improved access and quality, community involvement, health literacy and broader determinants of health. The primary health care Theory of Change provides a framework for monitoring the progress of the SDGs and the objectives of universal healthcare.^10^ It aims to connect the multiple components of primary health care to their desired outcomes. Figure 2 demonstrates the conceptual framework of a functional primary health care system.

## Practical examples of how family physicians and primary care teams can leverage the health systems thinking approach

### Leadership

Family physician leadership involves adaptable skills that can integrate into various roles. It includes empowering individuals to make autonomous decisions and providing precise feedback. Complex adaptive, situational and collaborative leadership styles are ideal for family physicians, and the widely utilised ‘I-we-it’ leadership model is part of the family physician training programmes. The ‘I’ domain is the foundation of this discipline. It focusses on the individual’s self-awareness and self-management and how one can change and develop their leadership behaviours and values. The ‘we’ domain is focussed on building solid relationships and networks that allow one to offer effective leadership. It emphasises skills such as communication, mentoring and coaching others. The ‘it’ domain focusses on understanding the health system’s context. This is very important for family physicians as it allows them to make informed decisions and improve their effectiveness.^11^

### Clinical governance and capacity building

**FIGURE 3:**
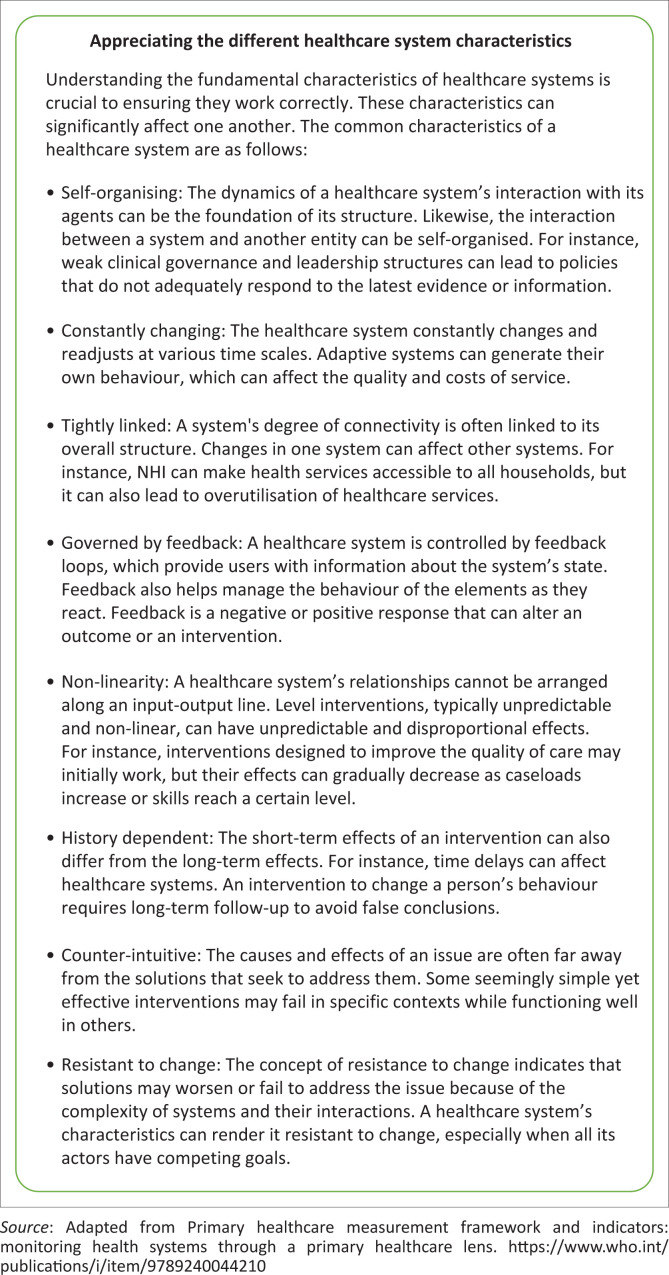
Different healthcare system characteristics.

Clinical governance in primary care is an essential responsibility of family physicians. It encompasses the leadership and implementation of processes to oversee tasks and facilitate cohesive teamwork. Critical considerations in clinical governance include teamwork, prioritisation and personal boundaries. In addition to disease-specific protocols, attention should be directed towards systematic issues such as care coordination across different healthcare system levels and patient satisfaction as demonstrated by health systems characteristics in Figure 3. Reflecting on collected data is integral for improving operational efficiency.^11^

Family physicians also contribute to the organisational culture by devising local policies and procedures that foster learning, reflection and accountability. Family physician training programmes in South Africa are best positioned to implement clinical governance in a resource-limited primary health care system.^24^

### Clinician-scholar

Practice-based research networks have been instrumental in developing primary care research programmes. They allow primary care clinicians and researchers to interact with one another and address critical issues related to the quality of care. In 2017, the Stellenbosch University Family Physician Research Network (SUFPREN) started such an initiative in collaboration with the district health services’ family physicians.^12^ It allows family physicians to participate in developing new research programmes as embedded clinician-researchers in the health system that address critical clinical governance issues and improve the quality of primary health care.

## Conclusion

Continuous improvement of the primary health care system requires ongoing monitoring and adaptation of strategies to meet evolving needs. This includes implementing new policies or strategies, initiatives for workforce development and community engagement. A thorough evaluation of the primary health care system is needed to identify areas of weakness and develop practical interventions to improve the system’s accessibility, effectiveness and fairness for all segments of society. Focussing on the building blocks of a primary health care system can enhance the quality of healthcare outcomes. Using a systems-based approach, collaboration between primary care providers, stakeholders and policymakers is essential to design and implement a resilient primary health care system that meets the population’s needs.
